# Pyroptosis-related long-noncoding RNA signature predicting survival and immunotherapy efficacy in patients with lung squamous cell carcinoma

**DOI:** 10.1007/s10238-024-01409-w

**Published:** 2024-07-03

**Authors:** Xiang Zhan, Jixian Li, Yi Ding, Fengge Zhou, Renya Zeng, Lingli Lei, Ying Zhang, Alei Feng, Yan Qu, Zhe Yang

**Affiliations:** 1 Tumor Research and Therapy Center, Shandong Provincial Hospital Affiliated to Shandong First Medical University, Jinan, 250021 Shandong China; 2 Tumor Research and Therapy Center, Shandong Provincial Hospital, Shandong University, Jinan, 250021 Shandong China

**Keywords:** Lung squamous cell carcinoma, Pyroptosis, LncRNAs, Tumor microenvironment, Risk model

## Abstract

Pyroptosis-related long-noncoding RNAs (PRlncRNAs) play an important role in cancer progression. However, their role in lung squamous cell carcinoma (LUSC) is unclear. A risk model was constructed using the least absolute shrinkage and selection operator (LASSO) Cox regression analysis based on RNA sequencing data from The Cancer Genome Atlas database. The LUSC cohort was divided into high- and low-risk groups based on the median risk score. For the prognostic value of the model, the Kaplan–Meier analysis, log-rank test, and Cox regression analysis were performed. A nomogram was constructed to predict the prognosis of patients, using a risk score and clinical parameters such as age, sex, clinical stage, and tumor node metastasis classification (TNM) stage. Afterwards, six common algorithms were employed to assess the invasion of immune cells. The Gene Set Enrichment Analysis (GSEA) was conducted to identify differences between patients at high and low risk. Furthermore, the pRRophetic package was employed to forecast the half-maximal inhibitory doses of prevalent chemotherapeutic drugs, while the tumor immune dysfunction and exclusion score was computed to anticipate the response to immunotherapy. The expression levels of the seven PRlncRNAs were examined in both LUSC and normal lung epithelial cell lines using RT-qPCR. Proliferation, migration, and invasion assays were also carried out to investigate the role of *MIR193BHG* in LUSC cells. Patients in the low-risk group showed prolonged survival in the total cohort or subgroup analysis. The Cox regression analysis showed that the risk model could act as an independent prognostic factor for patients with LUSC. The results of GSEA analysis revealed that the high-risk group showed enrichment of cytokine pathways, Janus tyrosine kinase/signal transducer and activator of the transcription signalling pathway, and Toll-like receptor pathway. Conversely, the low-risk group showed enrichment of several gene repair pathways. Furthermore, the risk score was positively correlated with immune cell infiltration. Moreover, patients in the high-risk category showed reduced responsiveness to conventional chemotherapeutic medications and immunotherapy. The majority of the long noncoding RNAs in the risk model were confirmed to be overexpressed in LUSC cell lines compared to normal lung epithelial cell lines by in vitro tests. Further studies have shown that downregulating the expression of *MIR193BHG* may inhibit the growth, movement, and infiltration capabilities of LUSC cells, whereas increasing the expression of *MIR193BHG* could enhance these malignant tendencies. This study found that PRlncRNAs were linked to the prognosis of LUSC patients. The risk model, evaluated across various clinical parameters and treatment modalities, shows potential as a future reference for clinical applications.

**Supplementary Information:**

The online version contains supplementary material available at 10.1007/s10238-024-01409-w.

## Introduction

Cancer is the leading cause of mortality worldwide. In 2022, lung cancer was diagnosed in approximately 2.5 million individuals, and the disease resulted in the deaths of around 1.8 million people [1]. It is the leading cause of cancer death in China [2]. Although there have been significant progressions in surgical techniques, chemotherapy, radiotherapy, molecular targeted therapy, and immunotherapy, the overall 5-year survival rate for lung cancer is less than 20% [3]. Hence, the effective management of lung cancer necessitates new advances in research.

Lung squamous cell carcinoma (LUSC) is responsible for around 30% of lung cancer cases worldwide, resulting in approximately 400,000 deaths annually [4, 5]. The clinical prognosis of LUSC is poorer than that of lung adenocarcinoma (LUAD), owing to the lack of targeted therapies [6]. At present, the prediction of the prognosis of LUSC mostly relies on the histopathologic diagnosis and tumor stage, although the accuracy is insufficient. Thus, there is a need for a more dependable prognostic model.

Pyroptosis is a type of cell death characterized by the release of inflammatory molecules in response to pathogenic microorganisms, chemotherapeutic drugs, radiation, or other stimuli. During pyroptosis, the cell membrane is perforated by the fragmented N-terminal of gasdermin (GSDM) proteins [7]. Pyroptosis contributes to the development, immunological microenvironment, treatment response, and prognosis of various forms of cancers [8].

Long-noncoding RNA (lncRNA) is a specific form of RNA that consists of more than 200 nucleotides and does not code for proteins [9]. It has an important role in the process of transcription, post-transcription, and epigenetic modifications in different diseases [10]. Pyroptosis-associated long non-coding RNAs (PRlncRNAs) are involved in the development of bladder cancer [11], LUAD [12], and gastric cancer [13]. However, the exact mechanism underlying the role of PRlncRNAs in LUSC is unclear.

This study aims to develop and validate a prognostic risk model for LUSC using PRlncRNAs. This model will be assessed for its ability to predict patient outcomes, its relationship with immune cell infiltration in tumors, and its potential to predict responses to chemotherapy and immunotherapy. The goal is to enhance personalized treatment strategies for LUSC patients.

## Materials and methods

### Data source

**Fig. 1:**
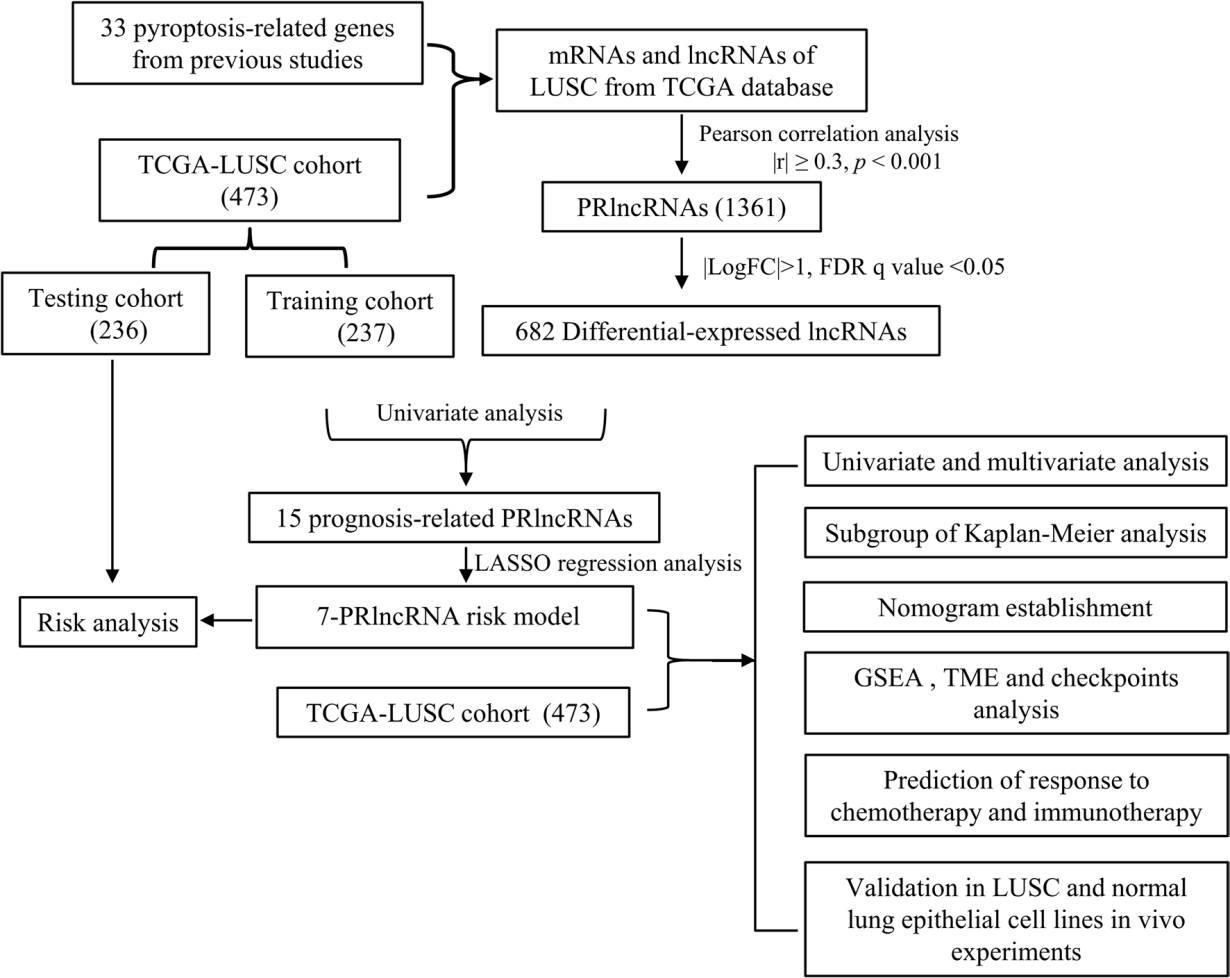
Study Design. TCGA, The Cancer Genome Atlas; LUSC, lung squamous cell carcinoma; PRlncRNAs, pyroptosis-related long-noncoding RNAs; FDR, false discovery rate; GSEA, Gene Set Enrichment Analysis; TME, tumor microenvironment

The RNA sequencing data and relevant clinical data of patients with LUSC were obtained from The Cancer Genome Atlas (TCGA) database, accessible at https://portal.gdc.cancer.gov/repository. Figure 1 shows the flowchart of the study design. Furthermore, a total of 33 pyroptosis-related genes (PRGs) were selected, including *ELANE, PJVK*, *TNF*, *NLRC4*, *NLRP3*, *NLRP7*, *GSDMA*, *CASP5*, *NLRP6*, *GPX4*, *PLCG1*, *PRKACA*, *SCAF11*, *CASP3*, *CASP4*, *GSDMD*, *NLRP2*, *AIM2*, *GSDMC*, *NOD2*, *TIRAP*, *GSDMB*, *NOD1*, *CASP6*, *CASP9*, *NLRP1*, *PYCARD*, *CASP1*, *CASP8*, *GSDME*, *IL18*, *IL1B*, and *IL6* [14–16].

### Identification of differentially expressed PRlncRNAs

To identify lncRNAs more accurately and rigorously, PRlncRNAs were classified as lncRNAs that were co-expressed with PRGs, with a Pearson's regression coefficient |r|≥ 0.3 and a *p*-value < 0.001 [17]. The selection of differentially expressed long non-coding RNAs (lncRNAs) involved comparing 49 normal tissues with 502 LUSC tissues using the limma package. The selection criteria were based on previous studies [18–22] and included the following criteria: |log fold change|> 1 and false discovery rate (FDR) < 0.05. An average was computed based on many data points collected from a single patient. Furthermore, the chi-squared test was performed to determine if the training and test cohorts were part of the larger cohort. In the training cohort, a univariate Cox analysis was performed to identify lncRNAs that had a significant prognostic value for LUSC.

### Construction of a risk model

The whole data set was randomly divided into two equal cohorts, namely the training cohort and the test cohort, using the createDataPartition function from the "caret" package https://github.com/topepo/caret/) [23]. The R "survival" package was used to perform univariate and multivariate Cox regression and survival studies. The software can be found at https://github.com/therneau/survival [24]. Only patients with overall survival > 1 month were selected for further investigations. With prognostic lncRNAs in the univariate Cox analysis, the LASSO Cox regression algorithm was applied to minimize the risk of overfitting using the “glmnet” package (https://cloud.r-project.org/package=glmnet) [25]. The risk model was developed by selecting seven lncRNAs. The following formula was employed to determine the risk score of each patient: risk score = Σi coefficient (lncRNAi) × expression (lncRNAi). The median risk score equitably divided the overall, training, and test cohorts into low- and high-risk groups. Finally, the Kaplan–Meier survival analysis was conducted, and risk curves were drawn. The "rms" program was employed to generate the nomogram.

### Gene set enrichment analysis

To better understand the specific biochemical pathways in the high- and low-risk groups, Gene Set Enrichment Analysis (GSEA) was performed using the KEGG gene sets and GSEA software (version 4.2.2).

### Immune landscape analysis

Six immune-related algorithms were used to describe the immune landscape in the two cohorts, including XCELL, TIMER, QUANTISEQ, MCPcounter, EPIC, and CIBERSORT-ABS [26, 27]. To have a comprehensive understanding of the relationship between the risk score and immunological status, Spearman's correlation analysis was performed to determine the correlation between the risk score and the infiltration of immune cells. Single-sample GSEA was used to examine immune cell activity, function, and pathway between the high- and low-risk groups. The immunological, stromal, and estimate scores for each patient were derived based on the composition of immune and stromal cells using the ESTIMATE algorithm. Furthermore, the Wilcoxon test was conducted to compare the levels of immune checkpoint molecules between the high- and low-risk groups.

### Association of the risk score with chemotherapy and immunotherapy responses

To identify the response of patients to chemotherapy drugs, the “pRRophetic” package was used to calculate the half-maximal inhibitory concentration (IC50) [28]. Higher IC50 values indicated lower sensitivity to drugs. The Wilcoxon test was used to assess differences between groups.

The Tumor Immune Dysfunction and Exclusion (TIDE) score was used as a biomarker of the efficacy of immunotherapy. Higher TIDE scores indicated poorer immunotherapy efficacy and patient prognosis [29]. The TIDE score was calculated using the TIDE database.

### RNA isolation and real-time reverse transcription quantitative polymerase chain reaction (RT-qPCR)

Total RNA was extracted using the RNAfast200 reagent kits (Fastagen, Thermo Fisher Scientific) from the H226, SKMES1, H520 LUSC cell lines, and the Beas-2B lung epithelial cell line. The PrimeScript™ RT reagent kit (TaKaRa) was used for reverse transcription. Table 1 presents the primer sequences for the seven lncRNAs. The expression levels of the lncRNAs *MIR193BHG*, *MIR3945HG*, *C10orf55*, *AC004069.1*, *LINC01956*, *AC008734.1*, and *AC090001.1* were normalized using GAPDH.

**Table 1:** Primer sequences of the seven lncRNAs

Ensemble ID	Primer’s name	Primers’s sequences (5' ~ 3')
ENSG00000262454	MIR193BHG-F	CGCCCAGCCAGGTTCAGATTTC
	MIR193BHG-R	ACTCTGCCTTCAAAGCCCATTAGC
ENSG00000251230	MIR3945HG-F	ATCAAATTAACTTTCCAGTCACCGC
	MIR3945HG-R	CCAGTCAGCAAATGTCGGAAG
ENSG00000251259	AC004069.1-F	ACAGAAGGATCAGAAGTAGATGGAA
	AC004069.1-R	TCCACAGAACAGGAACTAAAATACC
ENSG00000258910	LINC01956-F	GGGTGACTGGACAAAGGAGGTTATG
	LINC01956-R	CAGATGTGGCAGGCAGCAAGTC
ENSG00000269300	AC008734.1-F	CCAGCCAGGCGTGACTTCAATC
	AC008734.1-R	TTGGTGACCGTGTTATTCCAGAGTG
ENSG00000258343	AC090001.1-F	GCCCATGAGGGAGAACACCAAAC
	AC090001.1-R	GCCAACAGAGGAAAGTAGCAGAAGG
ENSG00000222047	C10orf55-F	GATTCGGGAGGAGGCTTCATCAAAG
	C10orf55-R	GCGAGAGGGAGAGGGAAGAGTG

### Cell culture and transfection

The human LUSC cell lines SKEMS, H226, and H520 were obtained from CoBier Bioscience Company (http://www.cobier.com). The SK-MES-1 cells were cultured in Minimum Essential Medium (MEM) containing 10% fetal bovine serum (FBS), 1% non-essential amino acids, and 1 mM sodium pyruvate. H226 and H520 cells were cultured in RPMI-1640 medium (Gibco, Thermo Fisher Scientific) and enriched with 10% FBS (Gibco, Thermo Fisher Scientific) and 1% penicillin–streptomycin antibiotic solution. The cells were incubated at 37 °C in a 5% CO2 humidified incubator.

H226 and H520 cell lines were transfected with *MIR193BHG*-specific and negative-control siRNA oligonucleotides, which were manufactured by Ribobio Company (www.ribobio.com), using the Lipofectamine RNAimax reagent (Invitrogen, Thermo Fisher Scientific). SiRNA oligonucleotides specific to *MIR193BHG* were synthesized based on the target sequence 5'-GGCTGGATCTGTAATGAAA-3'. The siRNA was used at a final concentration of 10 μM.

After transfection for 48 h, the cells were collected for further experiment. The overexpression of *MIR193BHG* was performed using plasmids synthesized by Shanghai GeneChem Company (www.genechem.com.cn) and the Lipofectamine 3000 Transfection Kit (Invitrogen, Thermo Fisher Scientific).

### Proliferation assay

Cells were seeded at a density of 5 × 10^3^ cells/well with five replicates per group in 96-well plates and cultured in a complete medium. For each well, 10 μL of CCK-8 solution and 100 μL of fresh culture medium were added on days 1, 2, and 3, followed by a 2 h incubation at 37 °C. Absorbance was read against 450 nm in a microplate reader (Bio-Rad).

#### Wound Healing, migration and invasion assay

For the wound healing assay, 2 × 10^5^ cells/well (three replicates per group) were plated into a six-well plate and cultured to reach confluence. After confluency, the monolayer of the cells was scratched with a sterile tip and washed with RPMI-1640 to remove the detached cells. The cells were incubated in RPMI-1640 and photographed at 0 h and 18 h post-wounding. The closure area of the wound was calculated as follows: migration area (%) = (A_0_ – A_18_)/A_0_ × 100%. Where A_0_ represents the area of the initial wound area, and A_18_ represents the wound area at 18 h.

For the transwell migration assay, LUSC cells were resuspended in serum-free RPMI-1640 medium, and 1 × 10^5^ cells were introduced into the upper chamber of the transwell inserts (8‑µm pore size; 6.5 mm diameter; Corning) with 100 μL RPMI-1640. Next, 600 μL RPMI-1640 containing 10% FBS was added to the lower chamber. Following a 24 h incubation, the cells in the upper chamber were fixed with pure methanol at room temperature for 30 min (min). The cells on the upper surface of the chamber were then removed by swabbing, and the migrated cells, which adhered to the lower surface of the membrane, were stained with 0.1% crystal violet (Sigma‑Aldrich) for 30 min.

The number of migrating cells was quantified using an inverted light microscope at a magnification of 100 × . The experiments were performed in triplicates. The protocol for the transwell invasion assay was similar to that of the migration assay, with the exception that the membrane was pre-coated with Matrigel (Corning) before the addition of the cell suspension.

#### Statistical analysis

The hazard ratio (HR) was calculated using both univariable and multivariable Cox regression analysis. The FDR was employed to assess the statistical significance of multivariate variables. Each group had three independent in vitro experiment replications. The unpaired t-test was used to compare groups in terms of in vitro experiments. All *P*-values are calculated using a two-tailed test. A *P*-value less than 0.05 and an FDR q-value less than 0.05 were considered as statistical significance. This study made use of R version 4.1.2.

## Results

### Identification of PRlncRNAs

**Fig. 2:**
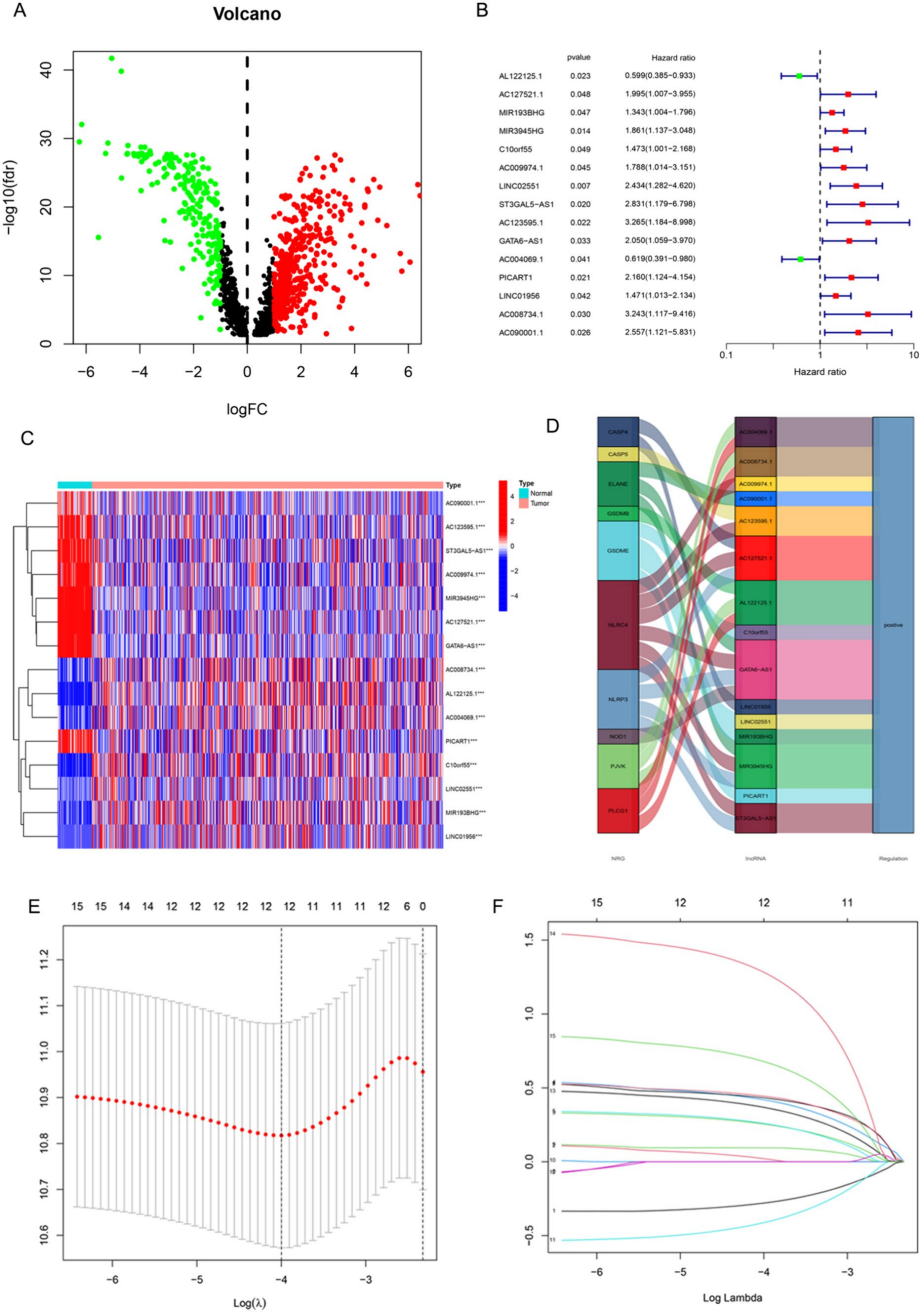
Identification of differentially-expressed PRlncRNAs and construction of the risk assessment model. Figure shows (**A**) Volcano plot of 467 upregulated (red) and 215 downregulated (green) PRlncRNAs in LUSC, (**B**) list of significant PRlncRNAs identified in the univariate Cox regression analysis of the training cohort (*P* < 0.05), (**C**) heatmap of the expression of significant lncRNAs in Fig. 2B between normal (bright blue) and tumor (orange) tissues (blue, low expression level; red, high expression level), ***P < 0.001. (**D**) An alluvial diagram illustrating the correlation between significant lncRNAs and PRGs, and (**E** and **F**) Cvfit and lambda curves showing LASSO regression with the minimum criteria

The correlation between PRGs and lncRNAs was examined to identify PRlncRNAs among the lncRNAs. Table 2 outlines the clinical characteristics of LUSC patients in the TCGA database. In total, 1,361 lncRNAs were identified as PRlncRNAs based on 33 PRGs, with a Pearson’s correlation coefficient |r|> 0.3 and *P* < 0.001. Furthermore, 215 PRlncRNAs were observed to be downregulated in LUSC tissues, while 467 were upregulated (|log fold change |> 1, FDR q < 0.05, Fig. 2A). Excel Supplementary 1 provides comprehensive data for the two groups. Out of the 682 lncRNAs that showed differential expression, only 15 were shown to have significant effects on the prognosis. Table 3 indicates that both the training and test cohorts were part of the larger overall cohort. Based on the univariate Cox analysis, it was shown that two lncRNAs, *AL122125.1* and *AC004069.1*, had a protective effect. However, the other 13 lncRNAs were identified as risk factors (Fig. 2B). Figure 2C displays the levels of expression of the 15 lncRNAs that are associated with prognosis. Figure 2D illustrates the detailed correlation between the 15 lncRNAs and PRGs.

**Table 2:** Clinicopathological characteristics of patients in TCGA-LUSC cohort

Characteristic	TCGA-LUSC cohort (n = 473)	
	Number	%
Age (years)		
< 60	90	19.0
≥ 60	378	79.9
Unknown	5	1.1
Gender		
Male	351	74.2
Female	122	25.8
Pathological stage		
Stage I	226	47.8
Stage II	156	33.0
Stage III	81	17.1
Stage IV	6	1.3
Unknown	4	0.8
T stage		
T1	108	22.8
T2	276	58.4
T3	68	14.4
T4	21	4.4
N stage		
N0	300	63.4
N1	125	26.4
N2	39	8.2
N3	5	1.1
NX	4	0.8
M stage		
M0	393	83.1
M1	6	1.3
MX	74	15.6
Outcome		
Alive	274	58.0
Dead	199	42.0

**Table 3:** The clinical characteristics of LUSC patients in the training and validation cohort

Characteristic	Training group No.	Training group No.	*P*_value
Age (years)			0.259
< 60	50	40	
≥ 60	185	193	
Unknown	2	3	
Gender			0.812
Male	177	174	
Female	60	62	
Pathological stage			0.237
Stage I	104	122	
Stage II	79	77	
Stage III	48	33	
Stage IV	3	3	
Unknown	3	3	
T stage			0.356
T1	52	56	
T2	146	130	
T3	28	40	
T4	11	10	
N stage			0.112
N0	141	159	
N1	66	59	
N2	26	13	
N3	3	2	
Unknown	1	3	
M stage			0.99
M0	194	199	
M1	3	3	
MX	40	34	
Outcome			0.324
Alive	132	142	
Dead	105	94	

### Construction of the risk assessment model

Risk model was constructed based on seven PRlncRNAs in the training cohort using the optimal penalty parameter for the LASSO model. Figure 2E and 2F show the cvfit and lambda curves, respectively. The risk score of each patient was calculated using the following formula: risk score = MIR193BHG*0.399790084174908 + MIR3945HG*0.817495127372367 + C10orf55*0.378419049121408 + AC004069.1*(-0.749145912408482) + LINC01956*0.446803653985047 + AC008734.1*1.48534201299929 + AC090001.1*1.0462963935057 (Note: The gene symbol represents the relative lncRNA expression in the TCGA database). The detailed information on the seven lncRNAs included in the risk model is depicted in Table 4.

**Table 4:** Detailed information of the seven PRlncRNAs included in the risk model

LncRNA		Ensemble ID	Coef	HR	HR.95L	HR.95H	*P*_value
MIR193BHG	ENSG00000262454		0.399790084	1.491512	1.10312	2.01665	0.009386
MIR3945HG	ENSG00000251230		0.817495127	2.26482	1.299921	3.945937	0.003902
C10orf55	ENSG00000222047		0.378419049	1.459975	0.994112	2.144151	0.053626
AC004069.1	ENSG00000251259		-0.74914591	0.47277	0.281871	0.792958	0.004523
LINC01956	ENSG00000258910		0.446803654	1.563307	1.031488	2.369325	0.035196
AC008734.1	ENSG00000269300		1.485342013	4.416476	1.611135	12.10653	0.00389
AC090001.1	ENSG00000258343		1.046296394	2.847087	1.158378	6.997637	0.022584

**Fig. 3:**
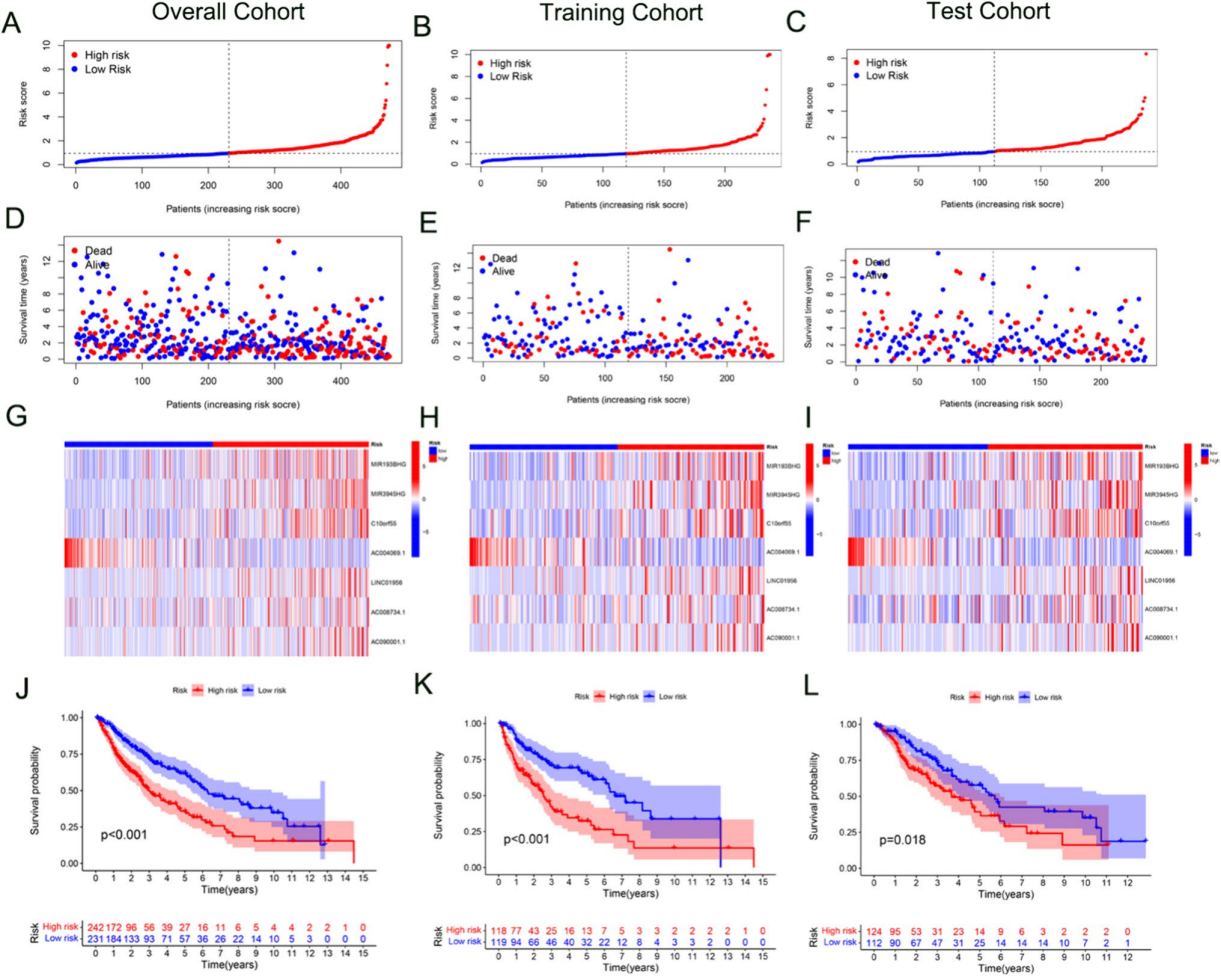
Effectiveness evaluation of the risk score. Figure 3 presents an evaluation of the effectiveness of the risk score in predicting patient outcomes across different cohorts. Panels (**A**, **B**, and **C**) illustrate the survival analysis, comparing the survival rates between patients in low-risk and high-risk groups for the overall, training, and test cohorts, respectively. Panels (**D**, **E**, and **F**) show the risk score distribution curves for the same groups across these cohorts, clearly distinguishing between the low-risk and high-risk categories. Heatmaps in panels (**G**, **H**, and **I**) depict the expression levels of model lncRNAs, highlighting differences in expression between the low-risk and high-risk groups within the overall, training, and test cohorts. Finally, panels (**J**, **K**, and **L**) present Kaplan–Meier survival curves, which further underscore the variation in survival outcomes based on lncRNA expression levels in both risk groups across the three cohorts

In the overall, training, and test cohorts, patients were divided into high- and low-risk groups according to the median risk score (Fig. 3A–C). The survival rate of patients in the low-risk group was significantly greater compared to those in the high-risk group (Fig. 3D–F). Figure 3G–I illustrate differences in the expression of lncRNA between the two groups. The majority of lncRNAs were enriched in the high-risk group. The Kaplan–Meier analysis demonstrated a significant correlation between the high-risk group and poor overall survival in all cohorts (Fig. 3J–L).

### Evaluation of PRlncRNA models

**Fig. 4:**
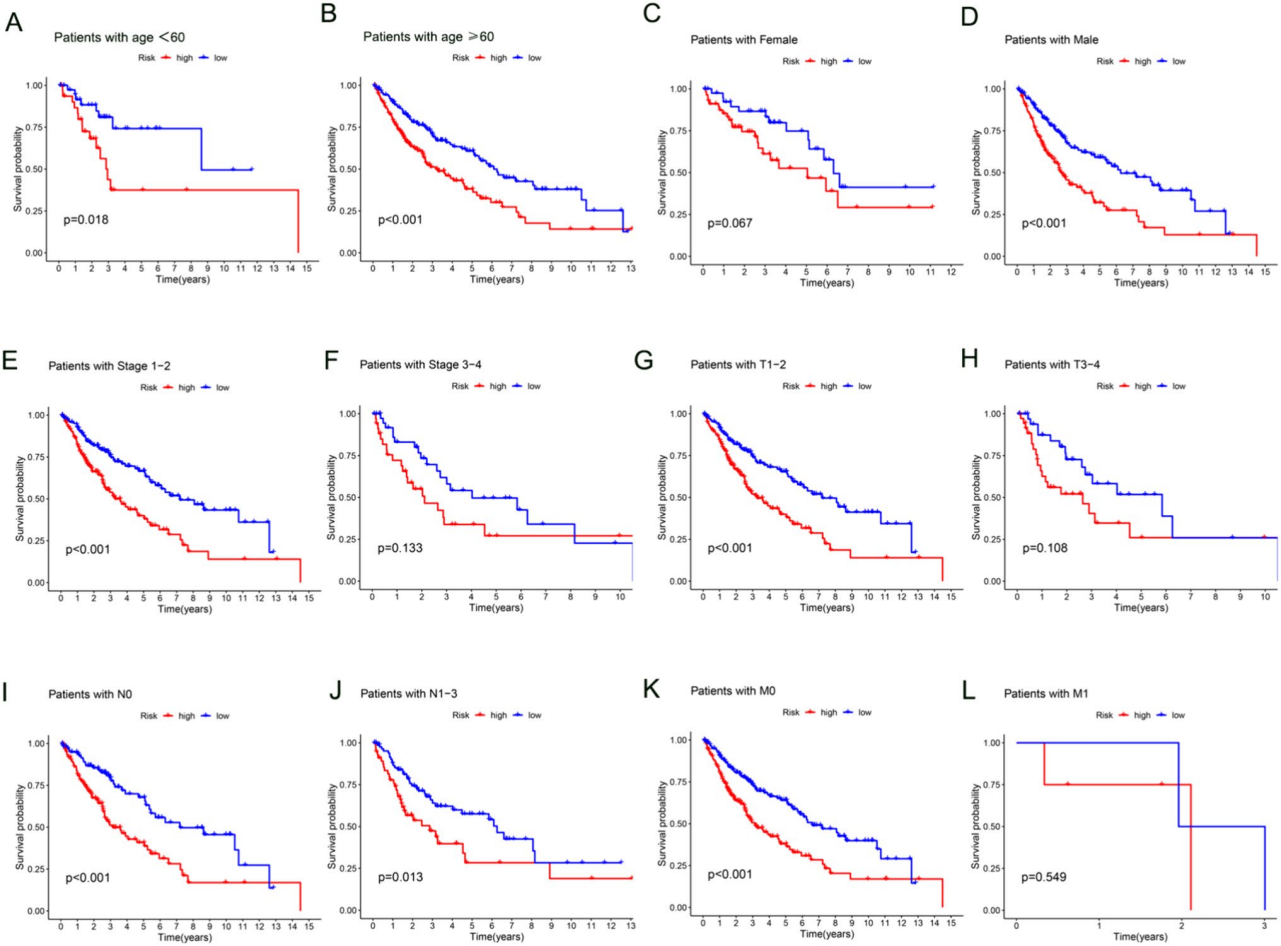
Kaplan–Meier analysis of overall survival. The figure shows the overall survival analysis by Kaplan–Meier in different subgroups: age (**A** and **B**), sex (**C** and **D**), histopathologic stage (**E** and **F**), T stage (**G** and **H**), N stage (**I** and **J**), and M stage (**K** and **L**)

Subgroup analyses were performed across various categories, including age, sex, histopathologic stage, and tumor node metastasis classification (TNM) stage. The survival rate differed significantly between the high- and low-risk groups in these analyses. However, there were no significant differences observed within individual subgroups, which may be due to the limited sample size (Fig. 4).

**Fig. 5:**
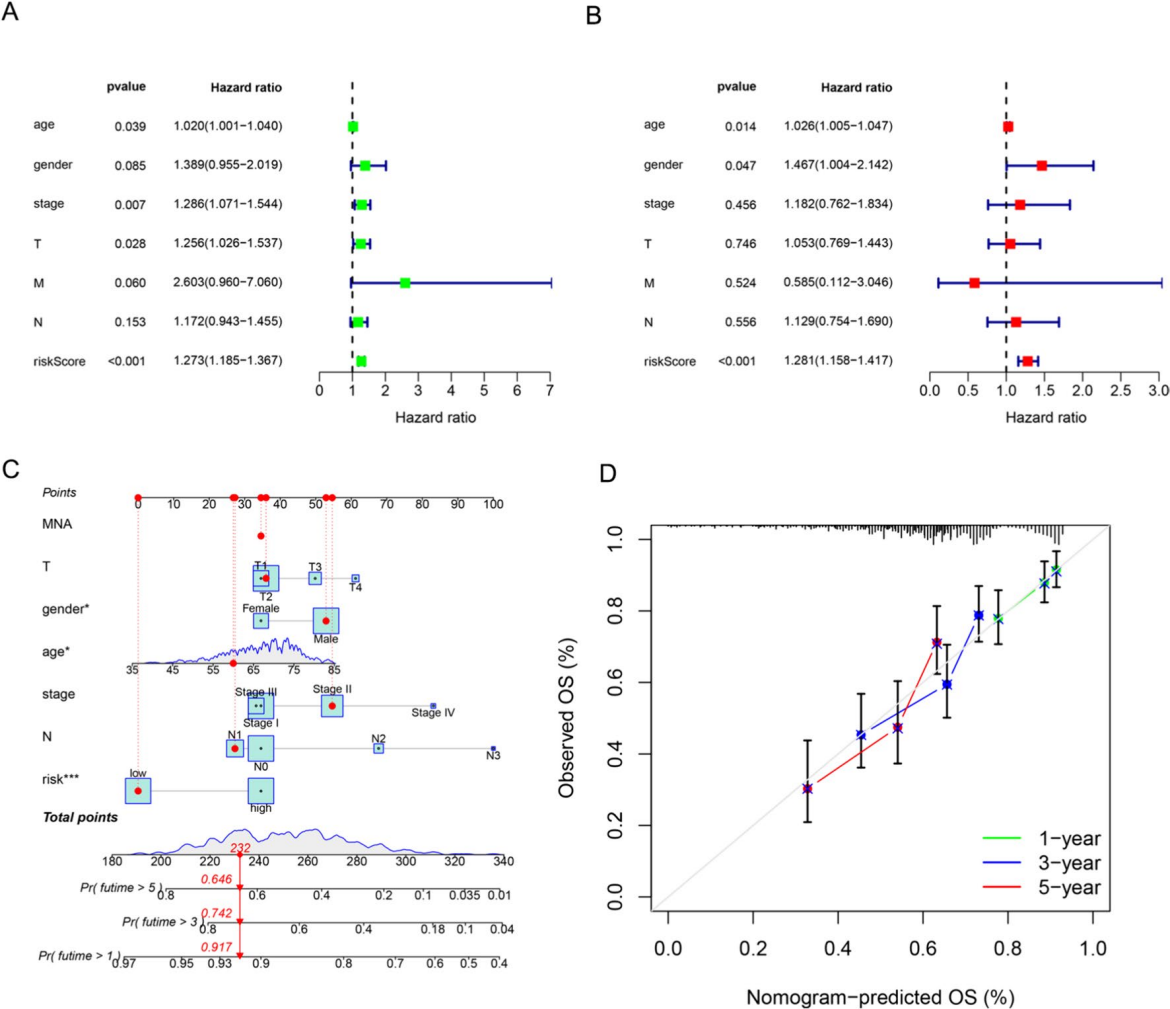
Correlation between the risk score and clinical characteristics. The figure shows (**A**) A univariate Cox regression study of clinical factors and patient risk scores, (**B**) A multivariate Cox analysis of clinical factors and patient risk scores, (**C**) A nomogram for the LUSC cohort that predicts 1-, 3-, and 5-year prognoses, and (**D**) Calibration plots of the nomogram for 1-, 3-, and 5-year survival

The univariate Cox regression analysis showed that age, histopathologic stage, T stage, and risk score were all associated with overall survival. A multivariate Cox regression analysis found that the risk score was an independent prognostic factor of LUSC (*P* < 0.001, HR = 1.273 [1.185, 1.367]) (Fig. 5A and B). The nomogram was constructed using age, gender, histopathologic stage, TNM stage, and risk score to predict 1-, 3-, and 5-year prognoses of LUSC (Fig. 5C and D).

### Discovery of molecular mechanisms using GSEA

**Fig. 6:**
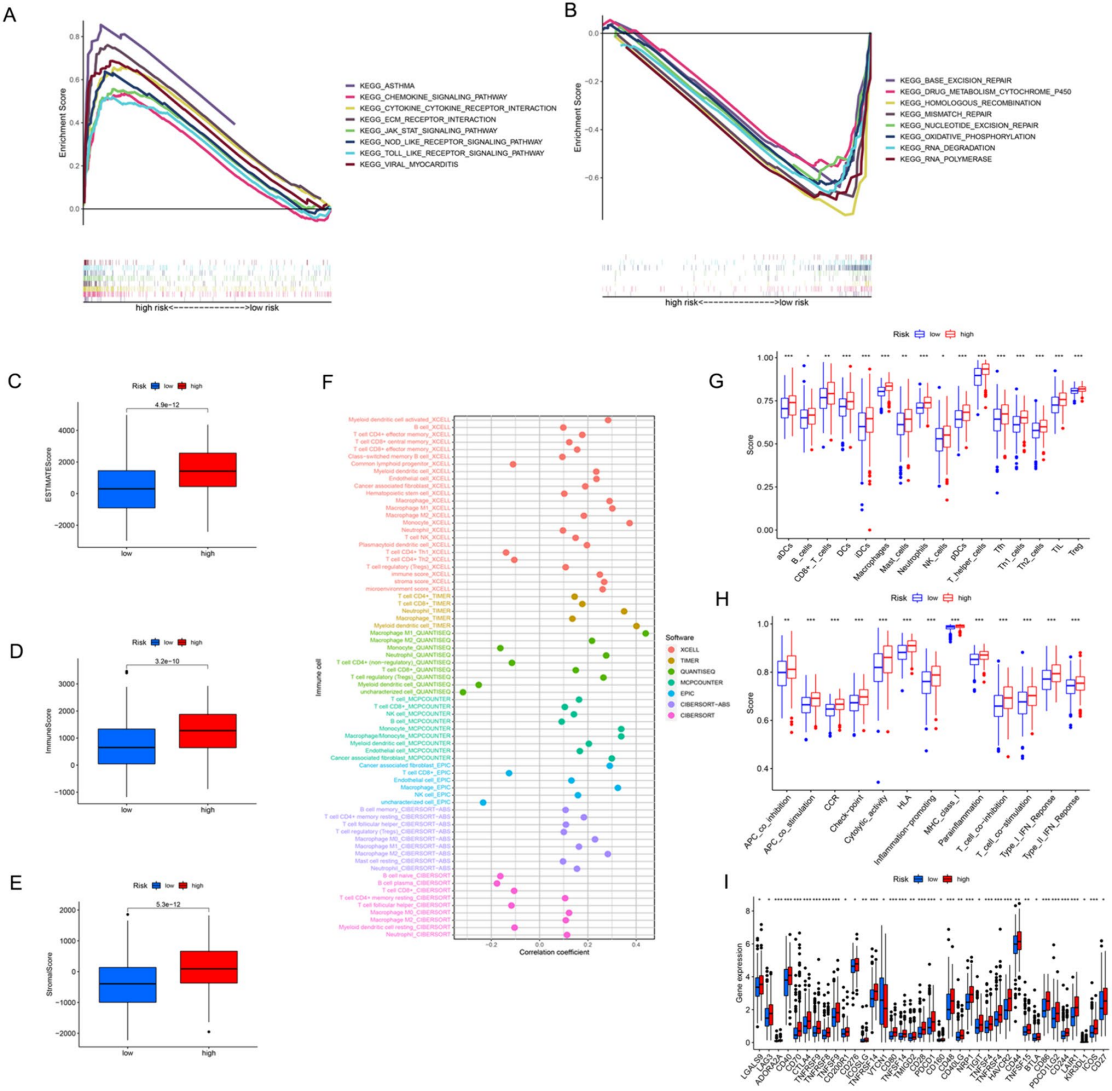
Comparison of molecular mechanisms, tumor microenvironment and checkpoints in high- vs. low-risk groups. (**A** and **B**) Pathway enrichment GSEA analysis of the low- and high-risk groups. (**C**–**E**) differences in the immune, stromal, and overall scores between the low- and high-risk groups, (**F**) correlation between immune cells and the risk score with different algorithms, (**G**) comparison of 16 immune cells in the low- (blue box) and high-risk (red box) groups, (**H**) comparison of 13 immune-associated pathways in the low- (blue box) and high-risk (red box) groups, and (**I**) box plots for comparison of immune checkpoint gene expressions between the low- and high-risk groups. *P < 0.05, **P < 0.01, and ***P < 0.001

GSEA was used to investigate the differences between high- and low-risk groups. Several cancer-related pathways, including the Janus tyrosine kinase/signal transducer and activator of transcription (JAK-STAT) signalling and Toll-like receptor (TLR) pathways, were more prevalent in the high-risk group. Immune-related pathways, such as the cytokine, cytokine-cytokine receptor, and viral myocarditis pathways, were also involved in the high-risk cohort. Many gene repair processes, including base excision repair, homologous recombination, mismatch repair, and nucleotide excision repair, were enriched in the low-risk group. The low-risk group had also an enrichment in the cytochrome P450 pathway, which is involved in drug metabolism (Fig. 6B).

### Comparison of tumor microenvironment and checkpoints in high- vs. low-risk groups

The immunological, stromal, and estimation scores were then assessed for the two groups. The high-risk group had a higher immunological score than the low-risk group (Fig. 6C–E). The majority of algorithms found a positive correlation between risk score and immune cell infiltration, including naïve B cells, CD4 + T cells, CD8 + T cells, dendritic cells, natural killer cells, and M0, M1, and M2 macrophages. In contrast, it had a negative correlation with Th1 and Th2 cells, as well as monocytes (Fig. 6F).

Immune cell infiltration was more in the high-risk group than in the low-risk group (Fig. 6G). Moreover, activation of all immune-associated pathways was enriched in the high-risk group as compared to the low-risk group (Fig. 6H). Furthermore, the expression of 37 immune checkpoint genes was up-regulated in the high-risk group than in the low-risk group (Fig. 6I).

### Predictive values in chemotherapy and immunotherapy

**Fig. 7:**
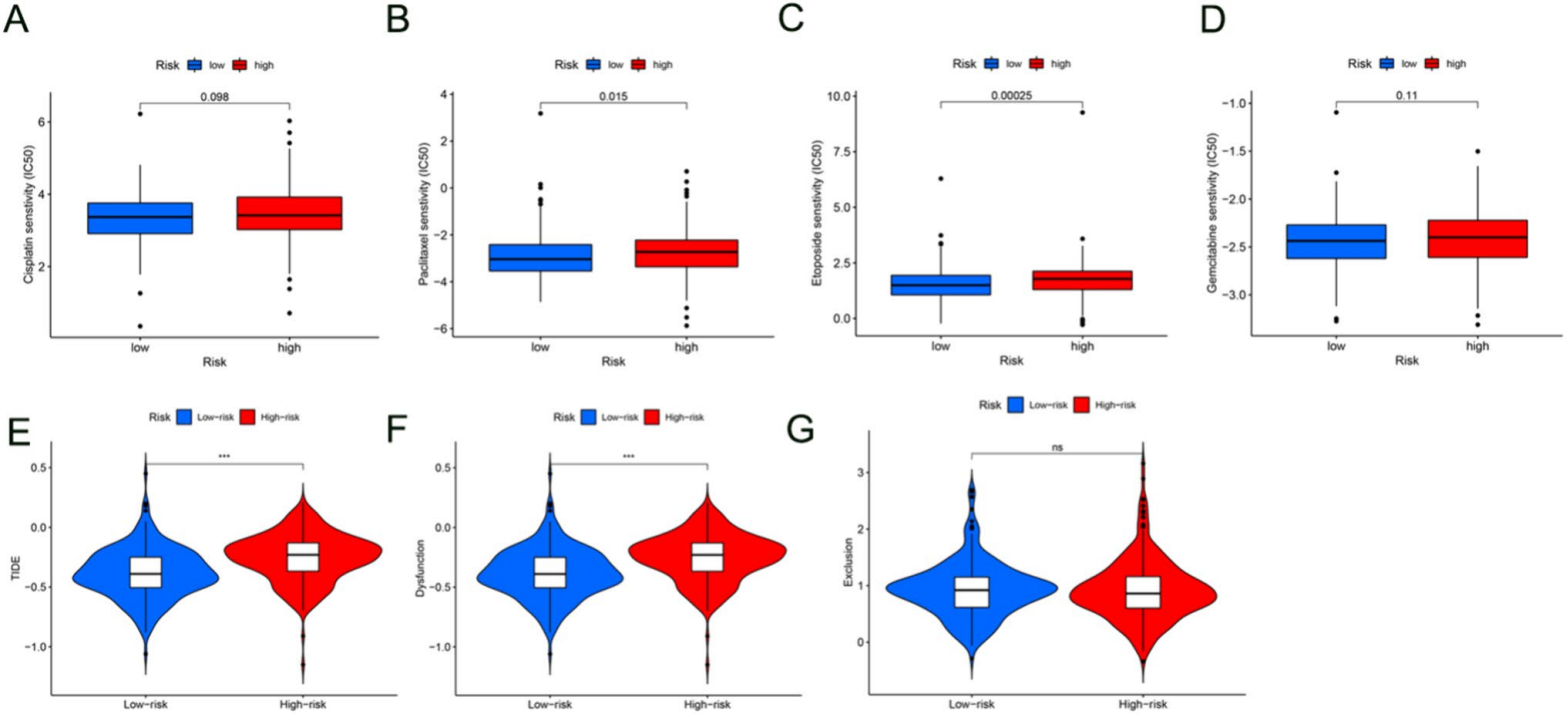
Predictive values in chemotherapy and immunotherapy. Figure shows (**A**–**D**) box plot presentation of differences in IC50 of four common chemotherapeutic drugs, i.e., cisplatin, paclitaxel, etoposide, and gemcitabine, for lung cancer between the low- and high-risk groups, (**E**–**G**) immune status based on TIDE and the grading system, including the TIDE score, the T Cell Dysfunction Score and the Exclusion Score

The “pRRophetic” package was used to assess the sensitivity of LUSC to four common chemotherapeutic drugs used for lung cancer: cisplatin, paclitaxel, etoposide, and gemcitabine, by calculating IC50. It was observed that patients with LUSC in the high-risk group were less sensitive to each of these chemotherapeutic drugs compared to those in the low-risk group (Fig. 7A–D).

Due to T cell dysfunction, the TIDE score was greater in the high-risk group than in the low-risk group (Fig. 7E–G), suggesting that patients with a high-risk score were prone to immunotherapy resistance.

### Validation in LUSC and normal lung epithelial cell lines

**Fig. 8:**
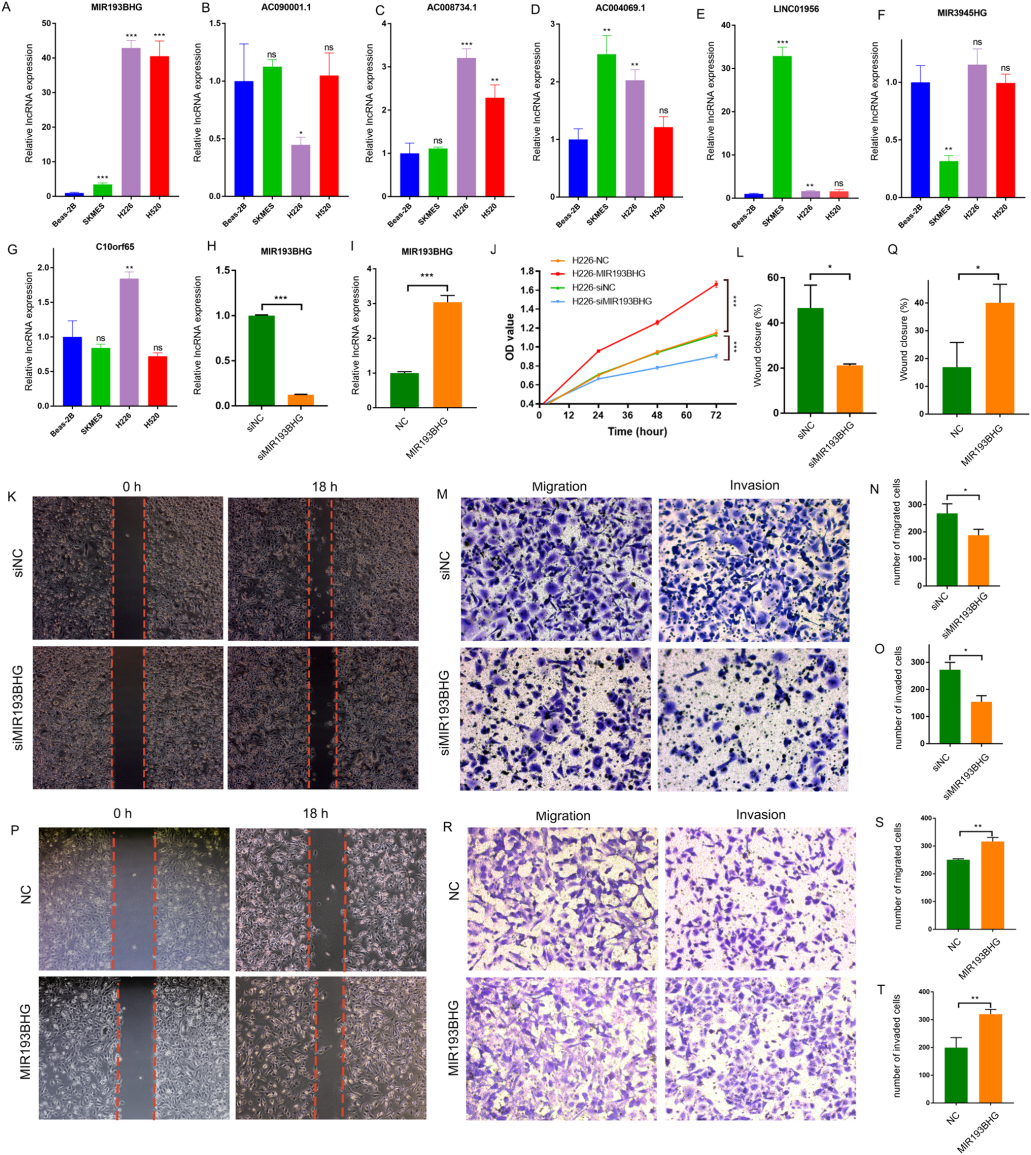
Validation in LUSC and normal lung epithelial cell lines. Figure represents (**A**–**G**) RT-qPCR of seven lncRNAs in LUSC and normal lung epithelial cells, (**H**) box plot of the *MIR193BHG* expression in control and siRNA-interfering H226 cells, (**I**) box plot of *MIR193BHG* expression in control and *MIR193BHG*-plasmid transfected H226 cells, (**J**) proliferation curve of H226 cells with varied expression of *MIR193BHG*, (**K**) Wound healing micrographs for control and siRNA-interfered H226 cells, taken at 0 h and 18 h post-scribing, (**L**) box plot for the wound healing assay analysis of control and siRNA-interfered H226 cells. (**M**) Migration and invasion assay’s representative images for control and siRNA-interfered H226 cells, (**N** and **O**) box plot representation of the migration and invasion assay in the control and siRNA-interfering H226 cells, (**P**) representative micrographs of wound healing assay for the control and *MIR193BHG*-plasmid transfected H226 cells photographed at 0 h and 18 h after scribing, (**Q**) Box plot representation of wound healing assay analysis in the control and *MIR193BHG*-plasmid transfected H226 cells, (**R**) representative micrographs of migration and invasion assay for the control and *MIR193BHG*-plasmid transfected H226 cells (**S** and **T**) Box representation of the migration and invasion assay analysis in the control and *MIR193BHG*-plasmid transfected H226 cells. *P < 0.05, **P < 0.01, ***P < 0.001

Three LUSC cell lines (SKMES, H226, H520) and one normal lung epithelial cell line (Beas-2B) were used to validate the findings. Following RNA extraction, RT-qPCR was performed to assess the expression levels of seven lncRNAs in each cell line. Expression of *MIR193BHG*, *C10orf55*, *AC004069.1*, *LINC01956*, and *AC008734.1* were up-regulated in the LUSC cell lines as compared to the Beas-2B cell line whereas *AC090001.1* and *MIR3945HG* were downregulated in the LUSC cell lines as compared to the Beas-2B cell line (Fig. 8A–G).

**Fig. 9:**
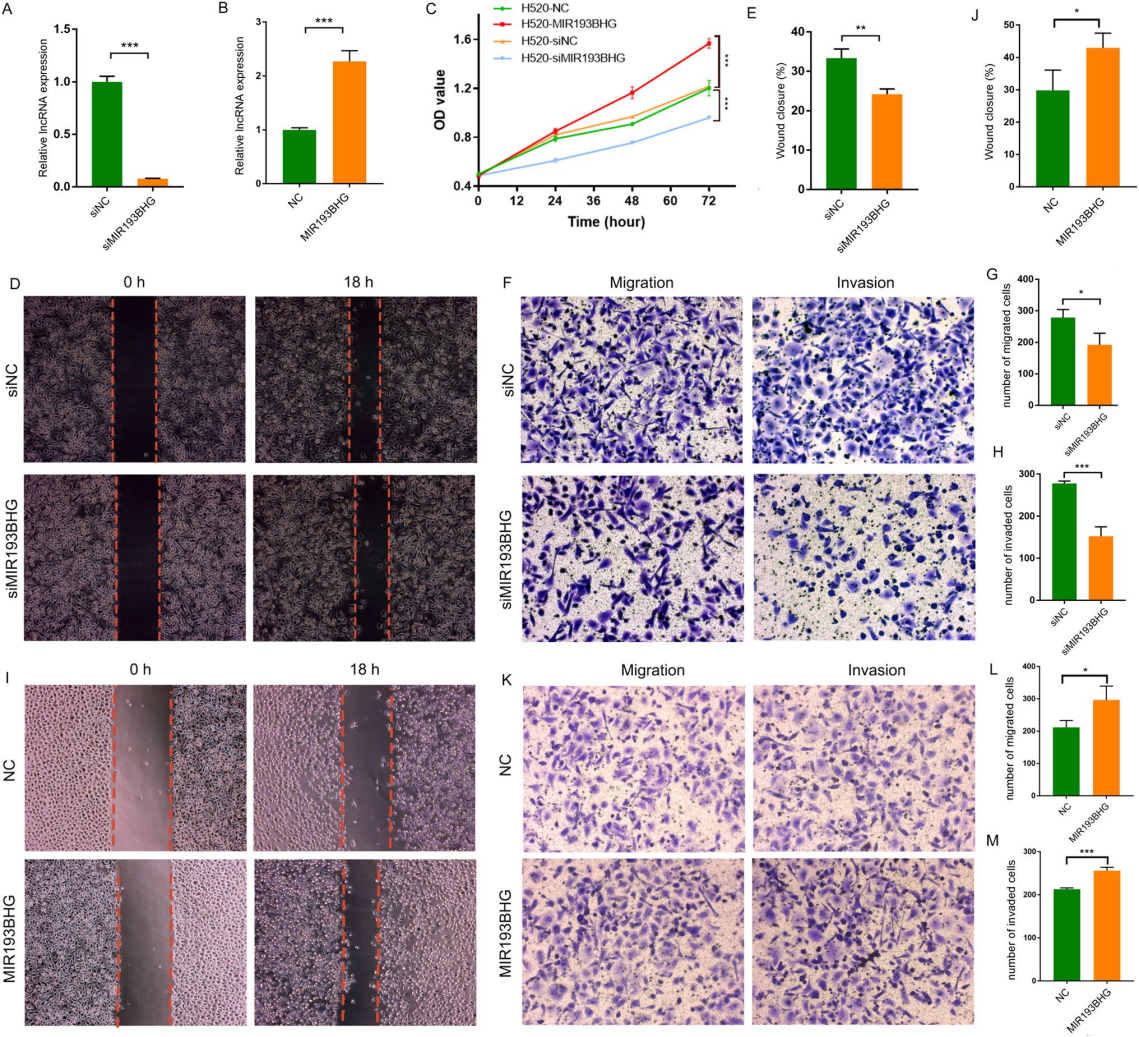
Validation in H520 cell lines. The figure shows (**A**) a box plot of the expression of *MIR193BHG* in control and siRNA-interfering H520 cells, (**B**) a box plot of the expression of *MIR193BHG* in control and *MIR193BHG*-plasmid transfected H520 cells (**C**) proliferation curve of H520 cells with different expression of *MIR193BHG*, (**D**) representative micrographs of wound healing assay for the control and siRNA-interfering H520 cells at 0 h and 18 h after scribing, (**E**) box plot representation of the wound healing assay analysis in the control and siRNA-interfering H520 cells, (**F**) representative micrographs of migration and invasion assay for the control and siRNA-interfering H520 cells, (**G** and **H**) box plot representation of the migration and invasion assay analysis in the control and siRNA-interfering H520 cells, (**I**) figures of wound healing assay for the control and *MIR193BHG*-plasmid transfected H520 cells photographed at 0 h and 18 h after scribing. (**J**) box plot of the wound healing assay analysis in the control and *MIR193BHG*-plasmid transfected H520 cells, (**K**) Images of migration and invasion assay for the control and *MIR193BHG*-plasmid transfected H520 cells, (**L** and **M**) box plot of the migration and invasion assay in the control and *MIR193BHG*-plasmid transfected H520 cells. *P < 0.05, **P < 0.01, ***P < 0.001

Following this, *MIR193BHG* was selected for validation due to its increased expression level among the seven lncRNAs. The data demonstrate that the expression of *MIR193BHG* was either reduced or increased in the H226 (Fig. 8H, I) and H520 (Fig. 9A and B) cell lines. Down-regulation of *MIR193BHG* resulted in the inhibition of cell proliferation in H226 (Fig. 8J) and H520 (9C) cells. The findings also revealed that the *MIR193BHG*-interfering group showed a decrease in migrated and invaded cells compared to the control group. This suggests that the ability of H226 (Fig. 8K–O) and H520 (Fig. 9D–H) cells to migrate and invade was significantly reduced after the knockdown of *MIR193BHG*. In contrast, the over-expression of *MIR193BHG* increased the capacities of H226 (Fig. 8P–T) and H520 (Fig. 9I–M) cell lines to proliferate, migrate, and invade. The findings indicated that *MIR193BHG* may serve as an oncogenic lncRNA that promotes tumor progression.

## Discussion

In this study, a PRlncRNA signature comprising seven lncRNAs, including *MIR193BHG*, *MIR3945HG*, *C10orf55*, *AC004069.1*, *LINC01956*, *AC008734.1*, and *AC090001.1*, was constructed. It may have the potential to enhance the accuracy of survival prognosis for patients with LUSC by differentiating between poor and good prognosis. The Cox regression study showed that the risk model outperformed conventional tumor features, such as T, N, and M stages, and might function as an independent prognostic indicator for patients with LUSC. Moreover, a nomogram was developed that incorporates these characteristics to accurately predict the probability of survival at 1, 3, and 5 years. This nomogram serves as a reference tool for evaluating the clinical prognosis.

In the present study,  the risk model consisted of seven PRlncRNAs. Among them, *MIR193BHG* plays a role in the regulation pathway of GPRIN1, which is associated with poor outcomes in kidney renal papillary cell carcinoma and LUAD [30]. The report validated the findings that *MIR193BHG* may act as a pro-tumor lncRNA in vitro by enhancing the proliferation, migration, and invasion of LUSC cells. *MIR3945HG* is among the top 10 lncRNAs that have the greatest diagnostic significance for LUSC. It distinguishes itself among the lncRNAs that are expressed differently in normal and malignant tissues [31]. *C10orf55* is among the four most significant genes correlated with plasminogen activator urokinase, a factor that is related to poor clinical results in head and neck squamous cell carcinoma [32]. While *AC004069.1* has been identified in tumor cells obtained from LUAD patients' xenografts, its precise function remains unclear [33]. *LINC01956* has been incorporated into a risk model for clear cell renal cell carcinoma [34], whereas *AC090001.1* was used in a survival prediction model for LUAD [35]. However, *AC008734.1* has not been documented in any published literature so far.

More than 50 types of cytokines use the JAK/STAT system to transmit signals, which in turn regulate hematopoiesis, trigger inflammation, manage the immunological response, and contribute to tumor progression. As "gatekeepers," TLRs guard the host against infections caused by bacteria, viruses, and other microorganisms. An imbalance in TLR signalling results in pathological states, including chronic inflammation, sepsis, asthma, autoimmune disorders, and cancer [36]. Aerobic glycolysis is a metabolic reaction in tumor cells. It is involved in tumor proliferation and correlated with DNA damage repair [37, 38]. A GSEA was performed to better understand the underlying mechanisms of the signature. The high-risk group showed enrichment of several cancer-related pathways, including chemokine, JAK-STAT, and TLR signalling pathways. The low-risk group showed enrichment of several gene repair signalling pathways, such as base excision repair, homologous recombination, and mismatch repair pathways.

Pyroptosis promotes the process of inflammation and the infiltration of immune cells by releasing a significant amount of chemokines. Zhang et al. discovered a positive correlation between GSDME-mediated pyroptosis and increased activation of natural killer cells, TAM cells, and CD8 + T cells in the tumor microenvironment of breast cancer and melanoma [39]. The study found a positive correlation between the risk score and the infiltration of various immune cells, including naive B cells, CD4 + T cells, CD8 + T cells, dendritic cells, natural killer cells, M0, M1, and M2 macrophages. On the other hand, there was a negative correlation between the risk score and the presence of Th1 and Th2 lymphocytes and monocytes. The prognosis of cancer varies depending on the different proportions of infiltrating immune cells [40]. In alignment with the previous studies, the present study indicated that the course of LUSC is influenced by the infiltration of immune cells. It was observed that the proportions of infiltrated immune cells can be altered by the presence of seven PRlncRNAs. However, the specific mechanisms remain unidentified. Additional research is necessary to elucidate the mechanisms in the future.

The results of the present study showed a significant correlation between the risk score and the expression of immune checkpoint genes. CD276 and PDCD1LG2 belong to the B7 transmembrane glycoprotein family. Their expressions are strongly associated with immune evasion and poor prognosis in non-small cell lung cancer (NSCLC) [41, 42]. This is also the case for other immune checkpoint genes [43–46]. The levels of immune checkpoint gene expressions were greater in the high-risk group compared to the low-risk group. Furthermore, patients who are at higher risk have a higher TIDE score, which suggests a reduced sensitivity to immunotherapy [29]. The results of this study showed that patients with a higher risk score had reduced sensitivity to commonly used chemotherapeutic medications such as cisplatin, paclitaxel, etoposide, and gemcitabine. These findings are significant for selecting appropriate treatments for the clinical treatment of patients with LUSC.

There are some limitations in this study. Initially, the risk model was developed and validated exclusively using the TCGA database due to the absence of appropriate data in other publicly available databases. Hence, future clinical investigations necessitate a bigger sample size to validate the model. Furthermore, due to the lack of comprehensive data in the TCGA database, it is challenging to incorporate genetic relationships into the present analysis. The role of *MIR193BHG* was investigated in LUSC cell lines, even though possible relationships between the risk score and immune cells, immunological function, immune checkpoints, chemotherapy response, and immunotherapy response were established. The detailed functions of the lncRNAs are still not fully understood.

## Conclusions

In conclusion, this study reveals a strong association between pyroptosis and LUSC. The risk model based on seven PRlncRNAs effectively predicts patient survival and outperforms traditional clinical factors. It also correlates with immune cell infiltration and predicts responses to chemotherapy and immunotherapy. These results highlight the potential of PRlncRNAs as biomarkers and guideposts for personalized treatment strategies in LUSC.

## Supplementary Information

Below is the link to the electronic supplementary material.

Supplementary file1 (XLSX 33 kb)

## Data Availability

The datasets presented in this study can be found in online repositories. (https://portal.gdc.cancer.gov/repository).

## Abbreviations

PRlncRNAs: Pyroptosis-related long-noncoding RNAs
LUSC: Lung squamous cell carcinoma
LASSO: Least absolute shrinkage and selection operator
TNM: Tumor node metastasis
GSEA: Gene set enrichment analysis
LUAD: Lung adenocarcinoma
lncRNA: Long-noncoding RNA
TCGA: The cancer genome atlas
TIDE: Tumor immune dysfunction and exclusion
FDR: False discovery rate
NSCLC: Non-small cell lung cancer
